# Candidate avirulent effector protein 2565 reduces clubroot incidence via rhizosphere microbiome restructuring and root exudate modulation

**DOI:** 10.3389/fmicb.2025.1614252

**Published:** 2025-07-07

**Authors:** Diandong Wang, Jingjing Liao, Jiayu Shi, Zhaoming Cai, Luyun Luo, Tailin Chen, Yixi Zhang, Xue Liang Tian

**Affiliations:** Yangtze Normal University, Chongqing, China

**Keywords:** tumorous stem mustard, *Plasmodiophora brassicae*, candidate avirulence effector, rhizosphere microbiome, root exudate

## Abstract

Clubroot disease caused by *Plasmodiophora brassicae* in tumorous stem mustard (*Brassica juncea* var. tumida) is difficult to control. Avirulent effector proteins can trigger plant immunity to fight pathogens, which exhibits promising prospect for managing this disease. Building on our earlier discovery that candidate avirulent effector protein 2565 reduces clubroot, this study used high-throughput sequencing and metabolomics to reveal the mechanisms at the microbiome and metabolome levels. Results showed that protein 2565 changed the beta diversity of rhizosphere bacterial communities and increased the abundance of Paenibacillus and Terrabacter. Additionally, this protein modified root exudates, leading to higher levels of disease-resistant metabolites like geniposidic acid and 8-hydroxyluteolin glucuronide. The close connection between microbial changes and metabolite shifts was confirmed by Procrustes analysis (*M*^2^ = 0.300, *p* = 0.001), indicating that protein 2565 both alters the root exudates and rhizosphere bacterial community to enhance plant defenses. Our findings highlight the potential of avirulent effectors in sustainable disease management through metabolic-microbial interaction.

## Introduction

Clubroot, a devastating disease affecting cruciferous crops, is caused by *Plasmodiophora brassicae* Woronin, an obligate biotrophic soil-borne protist. This pathogen induces profound morphological and physiological changes in host plants, resulting in the formation of characteristic root galls termed clubroots. *P. brassicae* exhibits a remarkably broad host range, infecting all 330 genera and 3,700 species within the Brassicaceae family, including economically vital crops such as rapeseed (*Brassica napus*), Chinese cabbage (*B. rapa* subsp. pekinensis), bok choy (*B. rapa* subsp. chinensis), kale (*B. oleracea* var. acephala), radish (*Raphanus sativus*), cauliflower (*B. oleracea* var. botrytis), and tumorous stem mustard (*B. juncea* var. tumida) (Zamani-Noor et al., 2022). In Fuling District, Chongqing, where tumorous stem mustard is a key commercial crop, annual yield losses exceeded 60% due to *P. brassicae* infection (Cai et al., 2023).

The life cycle of *P. brassicae* comprises three distinct phases: soil survival, primary root hair infection, and secondary cortical infection (Kageyama and Asano, 2009). During the soil survival phase, resting spores persist as dormant structures until favorable conditions trigger their germination. These spores then release biflagellate primary zoospores, which exhibit chemotactic migration toward host roots to initiate infection (Liu et al., 2020). Successful colonization by *P. brassicae* critically depends on the pathogen’s ability to actively suppress plant immune responses throughout the infection process.

Plant pathogens employ diverse effector proteins to suppress host immunity and facilitate infection (Hossain et al., 2025). *P. brassicae* secretes effector-like proteins that play vital roles in infection. Recent studies have functionally characterized specific effectors active during early and late infection stages (Chen et al., 2019; Pérez-López et al., 2020; Xu et al., 2025). These effectors exhibit multifunctional properties, including induction of host cell death and H_2_O_2_ accumulation, suppression of chitin-triggered immunity, inhibition of jasmonic acid, ethylene, and salicylic acid signaling pathways, promotion of root enlargement (Chen et al., 2019; Pérez-López et al., 2020).

Notably, when a pathogen-derived effector is recognized by the host, it transitions into an avirulent effector, triggering plant resistance and reducing infection efficiency. In previous work, we identified a candidate avirulence secretory protein (2565) from *P. brassicae* that reduced clubroot incidence and altered the rhizosphere microbiome (Shi, 2021). To uncover the potential mechanisms of protein 2565-mediated microbial restructuring and its interaction with root exudates, we combined high-throughput sequencing and metabolomic method to analyze the rhizosphere bacterial community and root exudates of tumorous stem mustard. Our findings enhance the understanding of clubroot pathogenesis and highlight the potential of avirulent effectors in sustainable disease management.

## Materials and methods

### Collection of *Plasmodiophora brassicae* resting spores in tumorous stem mustard

*P. brassicae* was isolated from the clubroot disease nursery of tumorous stem mustard at Yangtze Normal University. Field-infected roots exhibiting clubroot symptoms were collected and processed as follows. Roots were soaked in 70% ethanol for 1 min and rinsed three times with sterile water. Subsequently, roots were homogenized in sterile water (1:5, w/v) using a tissue homogenizer at low speed. The homogenate was filtered through a 100 μm sieve to remove large plant debris. The filtrate was mixed with 60% sucrose solution (1:1 v/v) and centrifuged at 3,000 × g for 20 min at 4°C. The pellet containing highly purified resting spores was collected from the bottom of the centrifuge tube. The pellet was resuspended in sterile water and centrifuged three times to remove residual sucrose. The purified spore suspension was stored for subsequent use.

### Impact of infection by *Plasmodiophora brassicae* on the rhizosphere microbiota of tumorous stem mustard

The tumorous stem mustard cultivar “Yong’an Xiaoye” was used in this study. Seeds were surface-sterilized with 70% ethanol for 3 min, rinsed three times with sterile water, and sown in pots containing soil collected from a corn field with no history of tumorous stem mustard cultivation or *P. brassicae* contamination. Ten seedlings were planted per pot. At the cotyledon full expansion stage, experimental groups were classified as follows: (1) Disease treatment group (P): seedlings were inoculated with *P. brassicae* resting spore suspension (1.0 × 10^8^ spores/mL); (2) Protein 2565 treatment group (P + 2565): 50 mL of crude protein extract was applied to rhizosphere soil 24 h prior to inoculation with equivalent concentrations of *P. brassicae* resting spores; (3) Blank control (Ck): plants were treated with sterile water without pathogen inoculation. All treatments were repeated three times under standardized greenhouse conditions (25°C ± 2°C, 70% relative humidity, 16/8 h light/dark cycle). Five weeks post-inoculation, seedlings were carefully uprooted to maintain whole root. Gall formation on root was investigated and the disease incidence and index were calculated. Rhizosphere soil adhering to the roots was collected for microbial DNA extraction. Roots were then washed thoroughly and used to collect root exudates as follows.

### DNA extraction, Illumina sequencing and analysis

Total soil genomic DNA was extracted from the collected soil (0.5 g) using the FastDNA^™^ SPIN Kit for Soil (MP Biomedicals, Santa Ana, CA, United States) following the manufacturer’s instructions, and the DNA concentration and quality were then assessed with a NanoDrop 2000 spectrophotometer (Thermo Scientific, Wilmington, DE, United States) and 1% agarose gel electrophoresis, respectively. The V3–V4 region of the bacterial 16S rRNA gene was amplified with the primer pair 338F (5′-ACTCCTACGGGAGGCAGCAG-3′) and 806R (5′-GGA CTA CHV GGG TWT CTA AT-3′) using a PCR thermocycler (ABI GeneAmp 9700, Foster City, CA, United States) (Peiffer et al., 2013). Equimolar amounts of purified PCR products from each DNA sample were pooled and sequenced using the Illumina MiSeq PE300 platform (San Diego, CA, United States) at Majorbio Bio Pharm Technology Co., Ltd. (Shanghai, China).

The raw 16S rRNA sequencing data were cleaned by quality control processing: initial quality filtering and adapter trimming were performed using fastp (v0.19.6) (Chen et al., 2018), followed by paired-end read merging with FLASH (v1.2.11) (Magoč and Salzberg, 2011). Chimeric sequences were subsequently removed using the software USEARCH (v11.0.667) (Edgar, 2010). The high-quality sequences was grouped in QIIME2 (v2021.4) (Bolyen et al., 2019) through sample-specific barcode matching. Operational taxonomic units (OTUs) were clustered at 97% identity using the UPARSE pipeline (Edgar, 2013). Taxonomic assignment of representative sequences for each OTU was performed using the RDP Classifier (v2.13) (Wang et al., 2007) with the SILVA 138 SSU Ref NR 99 database.^1^

Alpha diversity including the Shannon and Simpson indices were calculated. Comparative analysis of bacterial taxonomic overlap between three treated groups was visualized through three-way Venn diagrams at class and genus levels. Taxonomic composition was also assessed at class and genus level.

For β-diversity, principal coordinate analysis (PCA) based on Bray–Curtis distance was calculated and plotted, using the R package vegan, to determine the main variable components in the rhizosphere bacterial communities (Dixon, 2003). The discrimination in bacterial community composition between groups was performed using analysis of similarities and analysis of dissimilarities (ANOSIM/adonis) with 999 permutations based on Bray–Curtis distance. The differentially abundant genera across the three treatment groups were identified using the linear discriminant analysis effect size (LEfSe) method (Segata et al., 2011), based on an LDA score threshold >3.0 and a Kruskal–Wallis test (*p* < 0.05).

### Root exudate collection

Root exudates were collected using the method (Vives-Peris et al., 2020). Briefly, the seedling roots were carefully washed to remove any remaining soil, during which time the roots were left as undamaged as possible, and dead roots were removed with sterilized steel tweezers. Ten biological replicates (individual plant roots) were subsequently immersed in 100 mL of sterile Milli-Q^®^ water within Pyrex^®^ conical flasks (250 mL capacity). The extraction system was maintained at 25 ± 0.5°C under ambient light conditions for 12 h using a precision incubator shaker. Each root exudate solution was filtered through a 0.22 μm millipore filter membrane to remove any root debris, flash-frozen in liquid nitrogen and then stored at −80°C until further metabolomic analysis.

### Metabolomic analysis of root exudates

The untargeted metabolomics analysis of root exudates was conducted using Liquid Chromatography-Mass Spectrometry (LC-MS) at Majorbio Bio Pharm Technology Co., Ltd. (Shanghai, China). Root exudates were thawed on ice, and homogenized by vortexing for 10 s. A 10 mL aliquot of the mixed sample was transferred to a 15 mL centrifuge tube, flash-frozen in liquid nitrogen, and dried to a powder using a vacuum freeze-dryer. Three hundred microliters of 70% methanol containing internal standards was added to each sample. The mixtures were vortexed for 3 min and centrifuged at 12,000 rpm (4°C) for 10 min, after which the supernatants were collected in autosampler vials. For quality assurance, equal volumes from each sample were pooled to create composite quality control (QC) samples. During LC-MS analysis, QC samples were injected at intervals of every 10 experimental samples. Metabolite identification was performed using the MetWare database (Metware Biotechnology Co., Ltd., Wuhan, China) with quantification achieved through multiple reaction monitoring (MRM) on a triple quadrupole mass spectrometer. Venn diagrams showing shared and specific metabolites between different groups were also plotted (Chen and Boutros, 2011). The PCA of primary metabolites based on Bray–Curtis distance was built using the R package vegan. Partial least squares discriminant analysis (PLS-DA) was conducted in the R ropls package, and the models were further evaluated with 200 permutations (Thévenot, 2025). Metabolites with (1) variable importance in the projection (VIP) ≥1, which was extracted from the PLS-DA result, and (2) fold change ≥2 and fold change ≤0.5 were regarded as differential metabolites. Volcano plots were generated using the ggpubr package in R to screen for differential metabolites between the different treatments through pairwise comparisons (Kassambara, 2023).

### Associations between rhizosphere bacterial communities and root exudates

To elucidate the interaction between rhizosphere bacterial communities and root exudates, Procrustes analysis was performed by aligning the principal component analysis (PCA) ordinations of differentially abundant bacterial genera and metabolites. Mantel tests based on Spearman’s correlation method were used to assess the global association between the rhizosphere bacterial communities (Ck, P, and P + 2565 groups) and differential metabolites. For insights into genus-metabolite interactions, Spearman’s correlation analysis was executed using the corrplot package in R (Wei and Simko, 2024), identifying significant associations (|*ρ*| > 0.4, *p* < 0.05) between differently bacterial genera and metabolomic features.

## Results

### Inhibitory effects of the protein 2565 on clubroot disease

**Figure 1:**
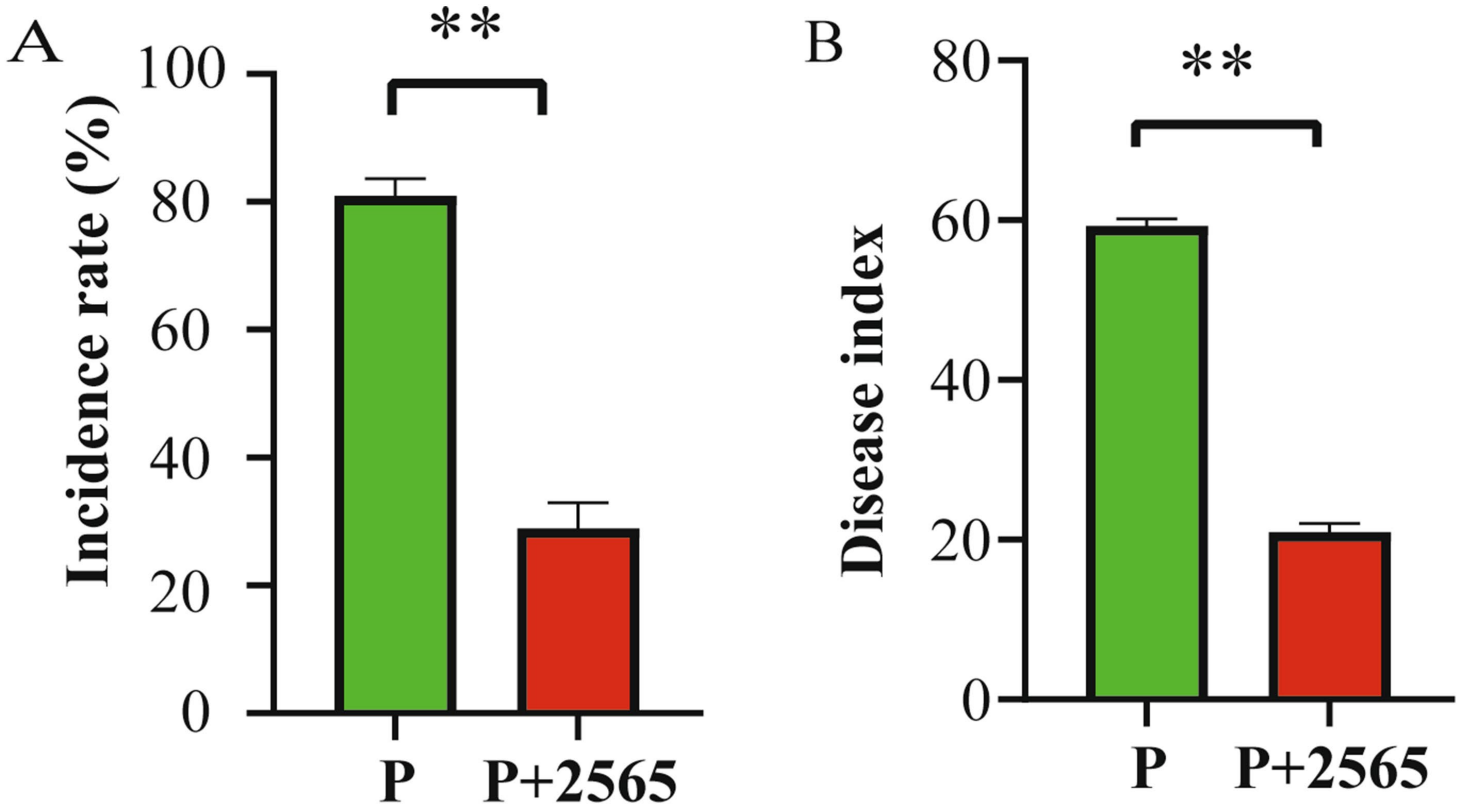
Inhibitory effect of the protein 2565 on clubroot disease. **(A)** Disease incidence rate. **(B)** Disease index. P: Inoculation with *P. brassicae* alone. P + 2565: Co-inoculation with *P. brassicae* and the 2565 crude protein. Data are presented as mean ± standard error (*n* ≥ 3). **Denotes highly significant differences between groups (*p* < 0.01).

Tumorous stem mustard inoculated only with *P. brassicae* displayed the highest disease severity, showing an 81.2% infection incidence and a disease index of 59.3. In contrast, co-inoculation of *P. brassicae* and the protein 2565 significantly suppressed disease, reduced the incidence rate (33.1%) and disease index (20.9), which was lower than those observed in the *P. brassicae*-only treatment (*p* < 0.05) (Figures 1A,B).

### Effects of the protein 2565 on rhizosphere bacterial communities

**Figure 2:**
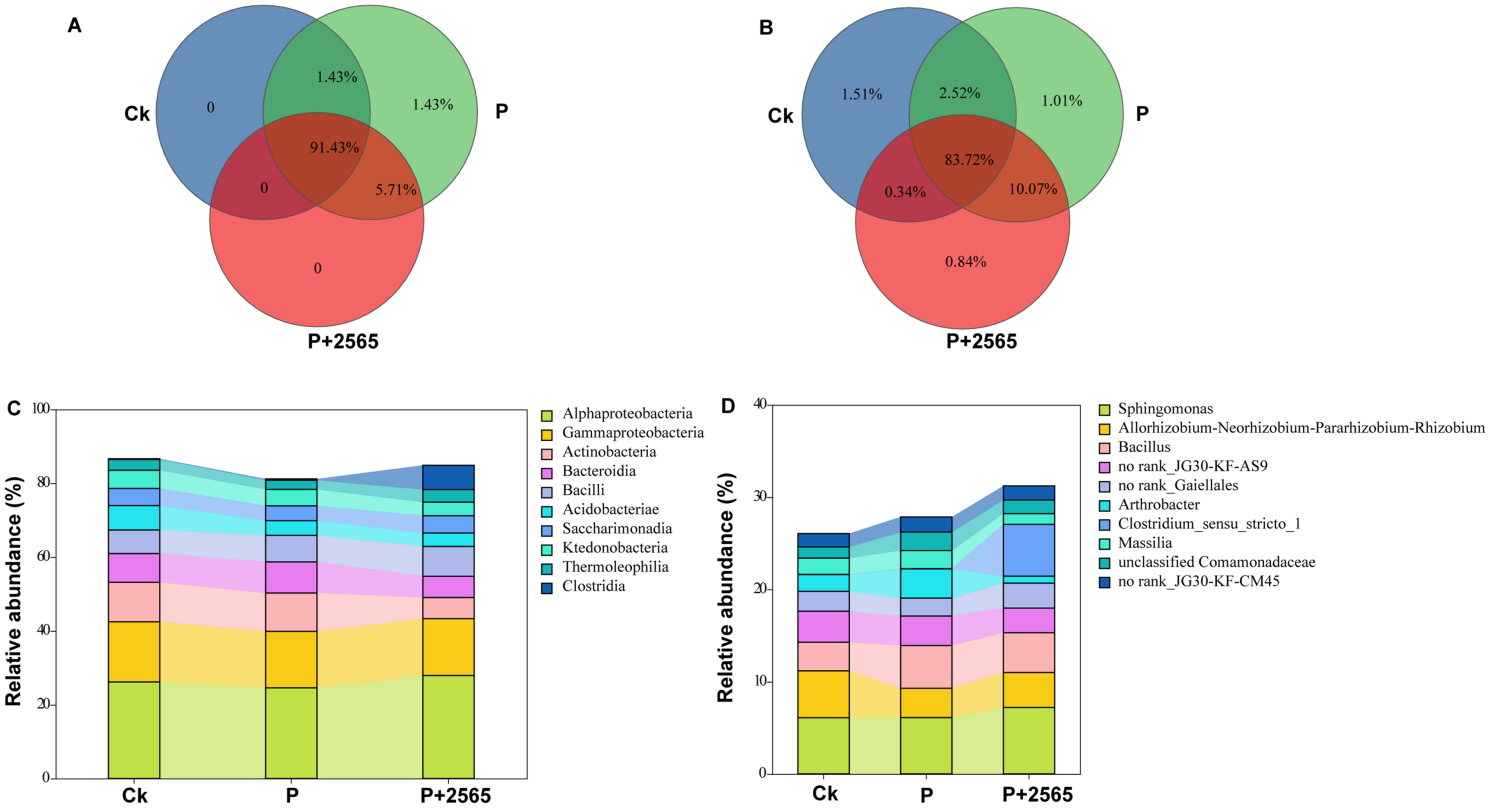
Characteristics of rhizosphere bacterial community structure under different treatments. **(A)** Class-level Venn diagram. **(B)** Genus-level Venn diagram. **(C)** Class-level bacterial community structure. **(D)** Genus-level bacterial community structure. P: Inoculation with *P. brassicae* alone. P + 2565: Co-inoculation with *P. brassicae* and the 2565 crude protein.

For alpha-diversity, the Shannon index and Simpson index of the rhizosphere bacterial communities of the three groups had no marked difference, respectively (Supplementary Figure S1). The Venn diagram analysis revealed that the proportion of shared bacterial taxa among all three treatment groups was 91.43% at the class level and 83.72% at the genus level (Figures 2A,B), indicating that the core bacterial communities remained relatively stable across the treatments. At the class level, Alphaproteobacteria and Gammaproteobacteria were the dominant bacterial groups across all three treatments, followed by Actinobacteria, Bacilli, and Bacteroidia (Figure 2C). Notably, the relative abundances of these classes exhibited differences among the three groups. At the genus level, *Sphingomonas* and *Bacillus* were identified as the most prevalent genera, while *Allorhizobium*-*Neorhizobium*-*Pararhizobium*-*Rhizobium* group and *Arthrobacter* exhibited secondary prevalence (Figure 2D). Similarly, the proportions of these genera varied significantly across the three treatments.

**Figure 3:**
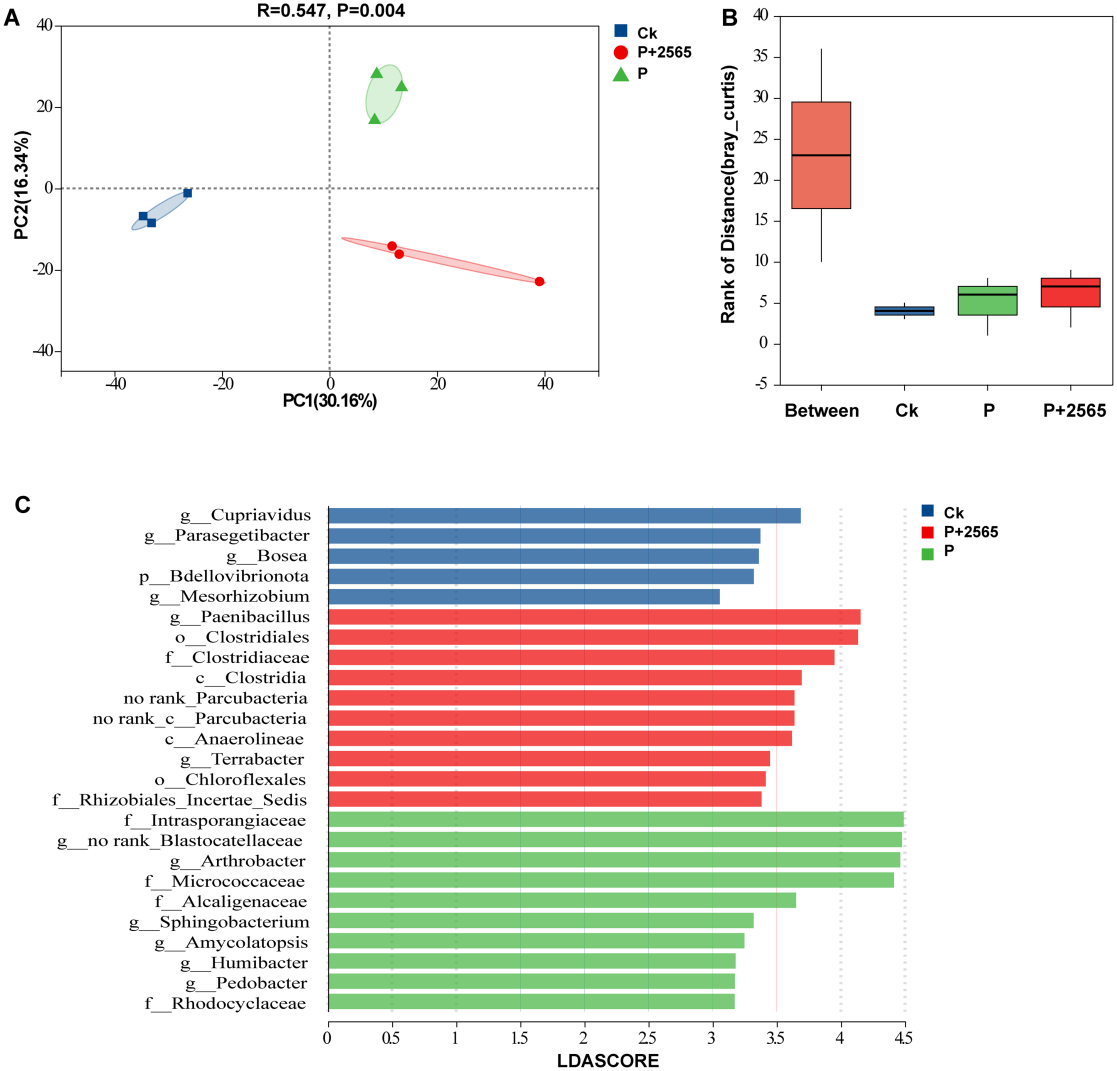
β-diversity of the rhizosphere microbial community. **(A)** PCA based on Bray–Curtis distance at the OTU level. **(B)** ANOSIM/Adonis analysis. **(C)** LEfSe analysis. Differentially abundant genera identified by LEfSe with a logarithmic (LDA score >3.0). P: Inoculation with *P. brassicae* alone. P + 2565: Co-inoculation with *P. brassicae* and the 2565 crude protein.

For beta-diversity analysis, PCA ordination was employed to determine the effects of various experimental factors on composition of rhizosphere bacterial communities. The PCA results revealed that marked dissimilarities between Ck and the other two groups (P and P + 2565). Principal components 1 (PC1, 30.16%) and 2 (PC2, 16.34%) collectively accounted for 46.5% of the total microbial community variation, highlighting significant structural divergence among the treatment groups (*R* = 0.5473, *p* = 0.004) (Figure 3A). Furthermore, inter-group distance box plots (based on OTU-level dissimilarities) demonstrated statistically distinct microbial community structures across P, P + 2565 and Ck (*p* < 0.05) (Figure 3B).

LEfSe analysis with logarithmic LDA >3.0 revealed that the signature taxa of Ck included *Cupriavidus*, *Parasegetibacter*, *Bosea* and *Mesorhizobium*. For P + 2565 group, the biomarker were *Paenibacillus* and *Terrabacter*. Additionally, *Arthrobacter*, *Sphingobacterium*, *Amycolatopsis*, *Humibacter* and *Pedobacter* were identified as distinctive taxa in P group (Figure 3C).

### Effects of the protein 2565 on root exudate composition

In total, 1,714 metabolites were detected, including amino acids, organic acids, fatty acids, nucleotides, lipids and other compounds (Supplementary Figure S2A). A total of 1,666 metabolites with percentage of 90.45% were shared by three treatment groups (Supplementary Figure S2B).

**Figure 4:**
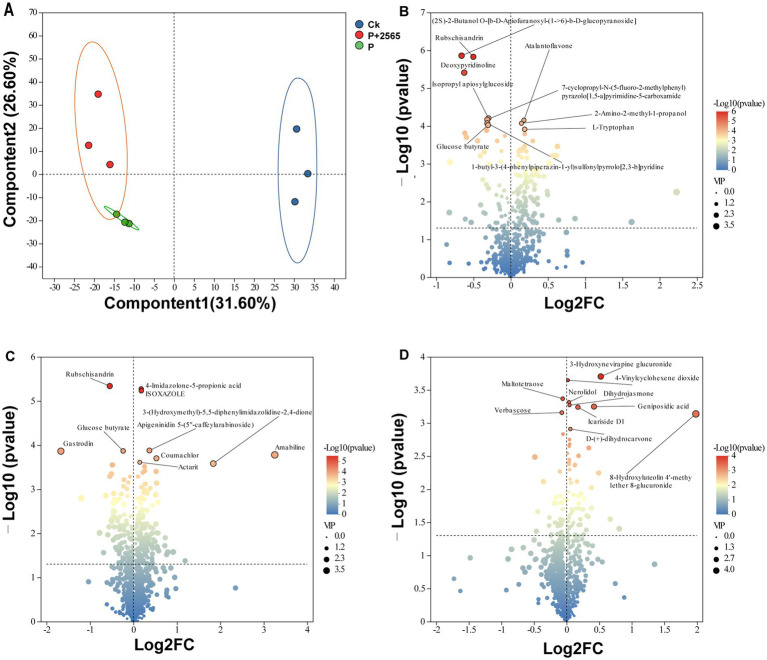
Changes in root metabolites of tumorous stem mustard under different treatments. **(A)** Metabolomic profiles of root exudates among three treatments assessed by PLS-DA. **(B)** The differential metabolites between Ck and P + 2565. **(C)** The differential metabolites between Ck and P. **(D)** The differential metabolites between P + 2565 and P. The top 10 differential metabolites with the largest and smallest fold changes are labeled with text.

According to the above findings, PLS-DA results revealed that samples were clearly separated in three groups (Figure 4A). Moreover, we constructed volcano plots using fold change and VIP to screen for differential metabolites among three experimental groups (Ck, P, P + 2565) through pairwise comparisons. In the Ck vs. P + 2565 comparison, Rubschisandrin, glucose butyrate, and gastrodin were significantly enriched in P + 2565 (*p* < 0.05, VIP >1.5), while 3-(hydroxymethyl)-5,5-diphenylimidazolidine-2,4-dione, 4-imidazolone-5-propionic acid, isoxazole, apigeninidin 5-(5″-caffeylarabinoside), amabiline, coumachlor, and actarit displayed pronounced accumulation in Ck (*p* < 0.01, VIP >2.0) (Figure 4B). For the Ck vs. P comparison, metabolites such as (2S)-2-butanol O-[*β*-D-apiofuranosyl-(1 → 6)-β-D-glucopyranoside], Rubschisandrin, and deoxypyridinoline were markedly upregulated in P. 7-cyclopropyl-N-(5-fluoro-2-methylphenyl) pyrazolo [1,5-a]pyrimidine-5-carboxamide and atalantoflavone exhibited significant enrichment in Ck (*p* < 0.01, VIP >1.5) (Figure 4C). For the P vs. P + 2565 comparison, metabolites enriched in P + 2565 contained geniposidic acid and 8-hydroxyluteolin 4′-methyl ether 8-glucuronide. Metabolites enriched in P included 3-hydroxynevirapine glucuronide, D-(+)-dihydrocarvone and 4-vinylcyclohexene dioxide (Figure 4D).

### Associations between rhizosphere bacterial communities and root exudates

**Figure 5:**
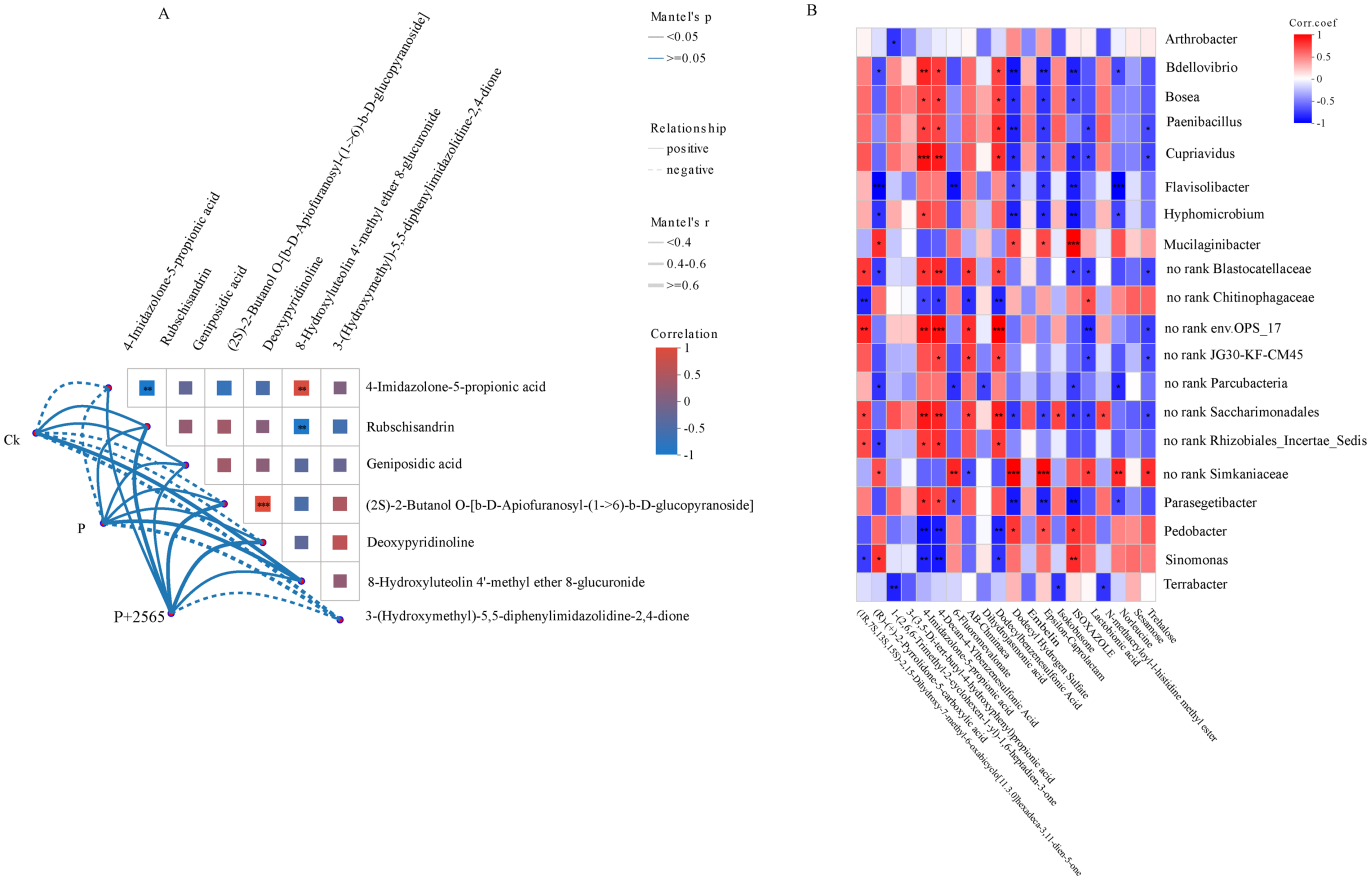
Multilevel association analysis between rhizosphere microbial communities and metabolites in tumorous stem mustard. **(A)** Global correlation between microbial communities and metabolites (Mantel tests). **(B)** Spearman correlation hot map of differential genera and key metabolites.

Procrustes analysis revealed a strong correlation between rhizosphere soil microbiome composition and root exudate metabolomic profiles across three groups (*M*^2^ = 0.300, *p* = 0.001) (Supplementary Figure S3), confirming significant interactions between rhizosphere bacterial communities and root-secreted metabolites. To further explore these relationships, Mantel tests were conducted between microbial community and differential metabolites, demonstrating that P + 2565 and P groups exhibited positive correlations with most differential metabolites, such as 4-Imidazolone-5-propionic acid, Rubschisandrin, Geniposidic acid, (2S)-2-Butanol O-[b-D-Apiofuranosyl-(1->66)-b-D-glucopyranoside], Deoxypyridinoline and 8-Hydroxyluteolin 4′-methyl ether 8-glucuronide (Figure 5A). In contrast, the Ck group showed negative correlations with the majority of these metabolites.

Spearman’s correlation analyses at the genus level revealed distinct microbial-metabolite interaction patterns (Figure 5B). *Bdellovibrio*, *Bosea*, *Paenibacillus*, and *Cupriavidus* exhibited significant positive correlations with 4-Imidazolone-5-propionic acid and 4-decan-4-ylbenzenesulfonic acid, while displayed strong negative associations with dodecyl hydrogen sulfate, *ε*-caprolactam, and isoxazole. In contrast, *Pedobacter* and *Sinomonas* demonstrated opposing trends, showing positive correlations with dodecyl hydrogen sulfate, ε-caprolactam and negative relationship with sulfonic acids.

## Discussion

### The protein 2565 alters rhizosphere microbial community structure

The study indicated that the candidate avirulent effector protein 2565 modifies the rhizosphere microbial community structure to some extent. From the diversity analysis, we found that the beta diversity of the bacterial communities of P and P + 2565 were significantly differ to the Ck, while the alpha diversity did not exhibited statistically discrimination. This discrepancy likely stems from the relatively short growth period of tumorous stem mustard after inoculation with *P. brassicae* (5 weeks post-inoculation), leaving insufficient time for significant rhizosphere microbial community restructuring. It further suggests that P + 2565-induced changes primarily occur at finer taxonomic or functional levels (e.g., beta diversity, metabolic pathways), which aligns with our significant beta diversity results (PERMANOVA, *p* < 0.05) and LEfSe/PICRUSt2 analyses. This observation is consistent with studies demonstrating that microbial functional shifts often precede structural changes and that alpha diversity frequently exhibits greater stability than beta diversity (Allison and Martiny, 2008; Hanson et al., 2012).

The distinct beta diversity of the bacterial communities among the P + 2565, P and Ck groups (ANOSIM, *R* = 0.547, *p* = 0.004), demonstrating that *P. brassicae* infection changed the bacterial communities of the rhizosphere of tumorous stem mustard. Previous studies demonstrated that *P. brassicae* infection alters the physiological state of plant roots, thereby modifying the community structures of endophytic bacteria and fungi (Wang et al., 2020; Tian et al., 2019). Such physiological changes may also lead to shifts in root exudates, further influencing rhizosphere bacterial composition. Similar phenomena have been reported in other soil-borne diseases, such as root-knot nematode infection and *Fusarium* wilt (Kudjordjie et al., 2024; Sun et al., 2024). Notably, the bacterial communities in the P + 2565 group and P group also exhibited distinct structural differences (PC2, 16.34%), suggesting that the protein 2565 modulates the rhizosphere microbiota of tumorous stem mustard. This phenomenon may align with the function of the Verticillium dahliae effector VdAMP2, which manipulates rhizosphere microbial communities (Snelders et al., 2020). However, the underlying mechanisms of manipulation differ significantly between the two proteins. Specifically, the VdAMP2 effector facilitates *V. dahliae* colonization through modulation of the rhizosphere microbiome. In contrast, effector 2565—an avirulence effector recognized by the host—blocks *P. brassicae* infection by similarly modulating the rhizosphere microbiome.

Importantly, the protein 2565 treatment significantly reduced the incidence and disease index of clubroot. We hypothesize that the disease-suppressive effects of the protein 2565 may partially stem from structural reorganization of the rhizosphere microbiome. LEfSe analysis further identified enrichment of genera such as *Paenibacillus* and *Terrabacter* in the P + 2565 group (Figure 3C). Paenibacillus, a well-characterized plant growth-promoting rhizobacterium, exhibits broad-spectrum antagonistic activity through the production of antimicrobial compounds (e.g., polymyxins and fusaricidins) and induction of plant systemic resistance (Dobrzyński and Naziębło, 2024; Daud et al., 2019). This genus may directly suppress *P. brassicae* in the rhizosphere of tumorous stem mustard, as exemplified by the biocontrol activity of *P. polymyxa* against clubroot disease in Brassica crops (Kang et al., 2024). These findings support its pivotal role in reshaping protective rhizosphere microbiomes during pathogen challenge. Similarly, *Terrabacter* is reported to have strong antimicrobial properties, potentially inhibiting *P. brassicae* (Akter et al., 2020).

### The protein 2565 alters the root exudate profiles

PLS-DA results demonstrated that *P. brassicae* infection significantly altered the root exudate profiles of tumorous stem mustard. These changes were likely caused by pathogens disrupting normal plant physiological processes, including disordered nutrient transport and impaired antioxidant system responses (Malinowski et al., 2012). Furthermore, pathogen infection induced tumor-like growths and skin breakage on roots, leading to significant leakage of cell contents (e.g., organic acids and amino acids). These leakages further modified the types and amounts of chemicals released from roots into the soil.

Volcano plot was used to demonstrate the distinct metabolic signatures among treatments. Rubschisandrin and deoxypyridinoline were markedly accumulated in the P group compared to the control group (Ck). Rubschisandrin, classified under phenylpropanoids and polyketides with broad-spectrum antimicrobial activity (Hou et al., 2016), is likely to enhance resistance against *P. brassicae* infection in tumorous stem mustard. Its accumulation reflects the activation of secondary metabolic defenses in tumorous stem mustard during early infection stages. Compared to P group, Geniposidic acid and 8-hydroxyluteolin 4′-methyl ether 8-glucuronide were significantly enriched in root exudates of the protein 2565 treated group. Geniposidic acid, an iridoid pathway intermediate, exhibits antioxidant activity by enhancing superoxide dismutase enzyme activity and gene expression while reducing reactive oxygen species and malondialdehyde levels (Wang et al., 2021). The flavonoid conjugate 8-hydroxyluteolin-4′-methyl ether-8-glucuronide exerts antimicrobial effects mediated by its antioxidant capacity (Chauhan et al., 2025).

The observed accumulation of antimicrobial compounds in the P + 2565 treatment group can be attributed to protein 2565-induced resistance responses in tumorous stem mustard. Functioning as a candidate avirulence effector, the protein 2565 exhibited secretory properties and suppressed clubroot progression in tumorous stem mustard (Shi, 2021). Once the protein 2565 recognized by host pattern recognition receptors of tumorous stem mustard, the defense cascades were active. This defense response could modulate root metabolic process, leading to the synthesis and accumulation of defense-related metabolites, which are ultimately released into the rhizosphere soil through root exudates. This highlights the avirulent effector characteristics of the protein 2565, which contrast with the virulence functions of PbEGF1, a canonical effector suppressing host immunity (Yang et al., 2024).

### Co-effect of root exudates and rhizosphere microbes on clubroot disease

The alternation of microbial communities may be driven by regulation of root exudates. Procrustes analysis confirmed a significant correlation between the microbiome and metabolome (*M*^2^ = 0.300, *p* = 0.001), suggesting that protein 2565 suppresses disease by regulating rhizosphere “metabolite-microbe” interactions. Multiple studies have demonstrated that root exudates are the primary factor influencing rhizosphere microbial community structure (Sasse et al., 2018; Olanrewaju et al., 2019), as they contain abundant nutrients such as sugars and amino acids that shape microbial composition. Root exudates and their interactions with rhizosphere microbiomes play a vital role in maintaining plant health (Afridi et al., 2024). When plants are infected by pathogens, they recruit beneficial microbes through specific root exudates to resist pathogen invasion (Gao et al., 2023; Xie et al., 2022). In this study, *Paenibacillus* was enriched in the rhizosphere of tumorous stem mustard following pathogen infection, particularly after the protein 2565 treated, indicating that the protein 2565 enhanced the pathogen-induced regulation of root exudation. These findings align with previous research demonstrating the efficacy of *P. polymyxa* as a biocontrol agent against clubroot disease in Brassica crops (Kang et al., 2024), supporting its role in rhizosphere microbiome during pathogen challenge.

Correlation heatmap analysis revealed significant positive associations between *Paenibacillus* and three metabolites: 4-Imidazolone-5-propionic acid, 4-decan-4-ylbenzenesulfonic acid, and dodecylbenzenesulfonic acid, suggesting these compounds may promote or recruit *Paenibacillus*. Mantel tests further identified a positive correlation between 4-imidazolone-5-propionic acid and the microbial community in the protein 2565 treated group, with *Paenibacillus* serving as the indicator species of this group. This strongly supports the critical role of 4-imidazolone-5-propionic acid in *Paenibacillus* enrichment. 4-imidazolone-5-propionic acid is a key intermediate metabolites in the histidine metabolic pathway. In *Bacillus subtilis*, 4-imidazolone-5-propionic acid is degraded to L-formiminoglutamic acid, a process essential for bacterial growth (Su et al., 2016). We speculate that 4-imidazolone-5-propionic acid may provide nutrients for the rapid growth of Paenibacillus species, thereby facilitating their colonization of the tumorous stem mustard root and enabling them to occupy a dominant ecological niche. Malic acid in watermelon root exudates induces motility in Paenibacillus species, enhancing its root colonization (Ling et al., 2011). In this study, it remains to be revealed whether 4-imidazolone-5-propionic acid can attract Paenibacillus species toward tumorous stem mustard roots. Future studies could employ *in vitro* supplementation experiments to assess the effects of this metabolite on *Paenibacillus* growth.

The reduction in clubroot disease incidence by the protein 2565 may involve two potential mechanisms. Firstly, pre-inoculation with the protein 2565 could active disease resistance in tumorous stem mustard, leading to the accumulation of defense-related metabolites (e.g., phytoalexins, pathogenesis-related proteins) in roots, thereby directly suppressing *P. brassicae* infection. Secondly, the protein 2565 induced immune response might regulate root exudate composition (e.g., flavonoids, organic acids), recruiting beneficial rhizosphere microbiota (e.g., *Paenibacillus*) to inhibit *P. brassicae* infection.

The next phase of research should focus on investigating the signaling pathways involved in the defense responses of tumorous stem mustard induced by the protein 2565, and evaluating the impacts of exogenous application of differential metabolites on both the rhizosphere microbial community structure and the incidence of clubroot disease caused by *P. brassicae*.

## Data Availability

The raw 16S rRNA reads have been uploaded to the NCBI Sequence Read Archive (SRA) database with the accession number PRJNA1251015.

## Footnotes

^1^ https://www.arb-silva.de

## Supplementary material

SUPPLEMENTARY FIGURE S1 Alpha diversity of three treated groups. **(A)** Shannon index. **(B)** Simpson index.

SUPPLEMENTARY FIGURE S2 Chemical characterization of root exudates across experimental groups. **(A)** Chemical class distribution of identified compounds. **(B)** Comparative Venn diagram illustrating compound overlap among treatment groups.

SUPPLEMENTARY FIGURE S3 Procrustes analysis between rhizosphere soil microbiome composition and root exudate metabolomic profiles.
