# Supplementary figures and images for: Candidate avirulent effector protein 2565 reduces clubroot incidence via rhizosphere microbiome restructuring and root exudate modulation
Source: Front Microbiol. 2025 Jul 7;16:1614252. doi: 10.3389/fmicb.2025.1614252 (PMC12277381; doi:10.3389/fmicb.2025.1614252)

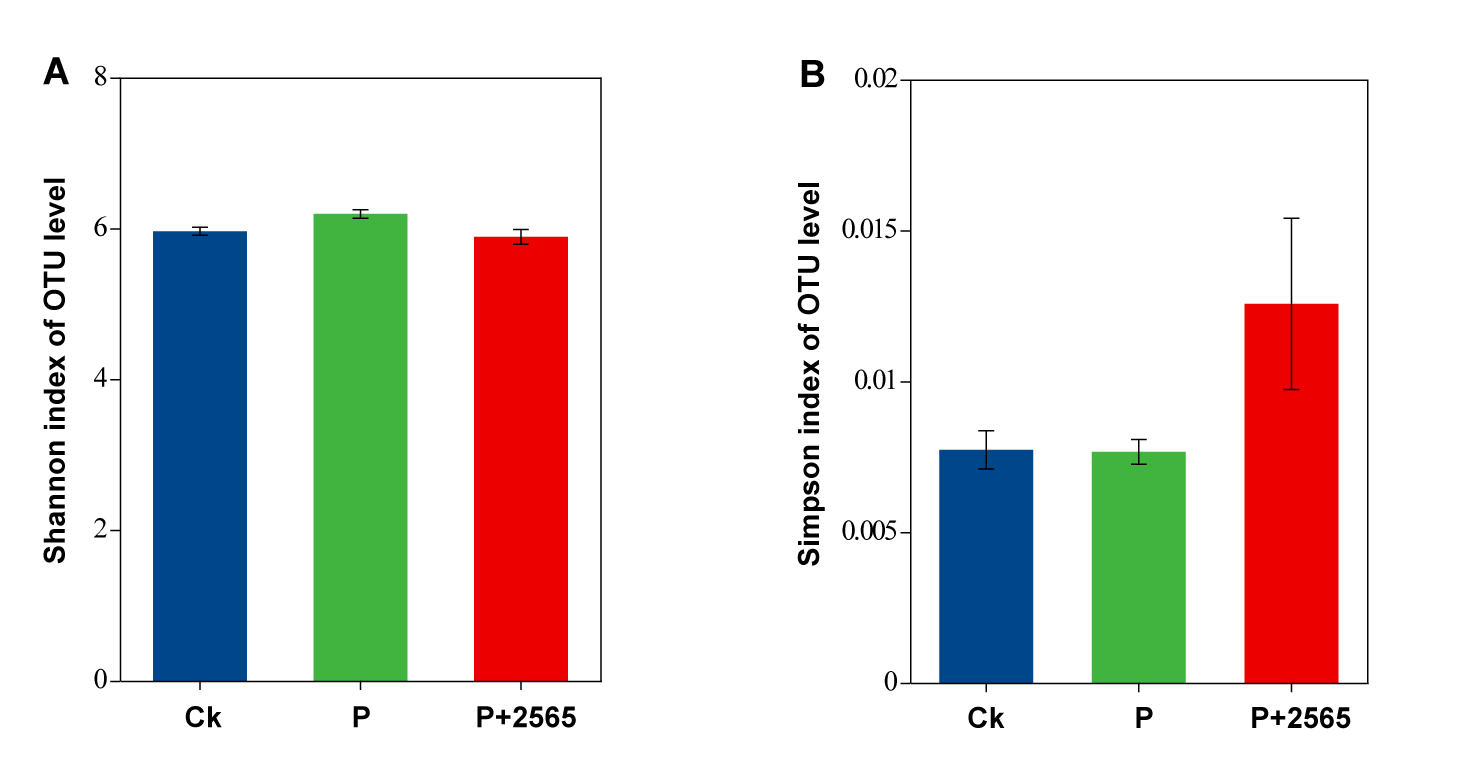

Supplement: SUPPLEMENTARY FIGURE S1 — Alpha diversity of three treated groups. (A) Shannon index. (B) Simpson index. [file Image_1.tif]

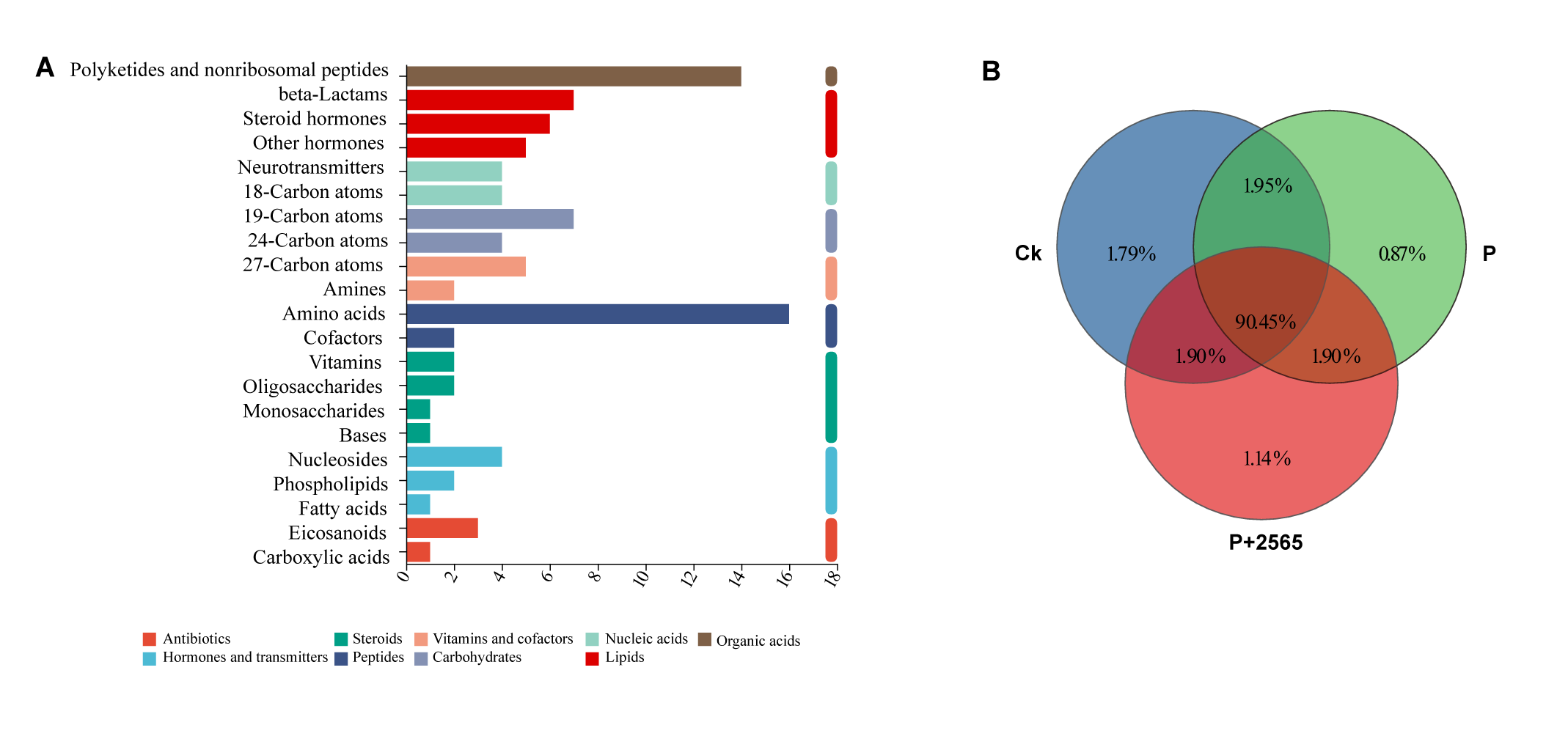

Supplement: SUPPLEMENTARY FIGURE S2 — Chemical characterization of root exudates across experimental groups. (A) Chemical class distribution of identified compounds. (B) Comparative Venn diagram illustrating compound overlap among treatment groups. [file Image_2.tif]

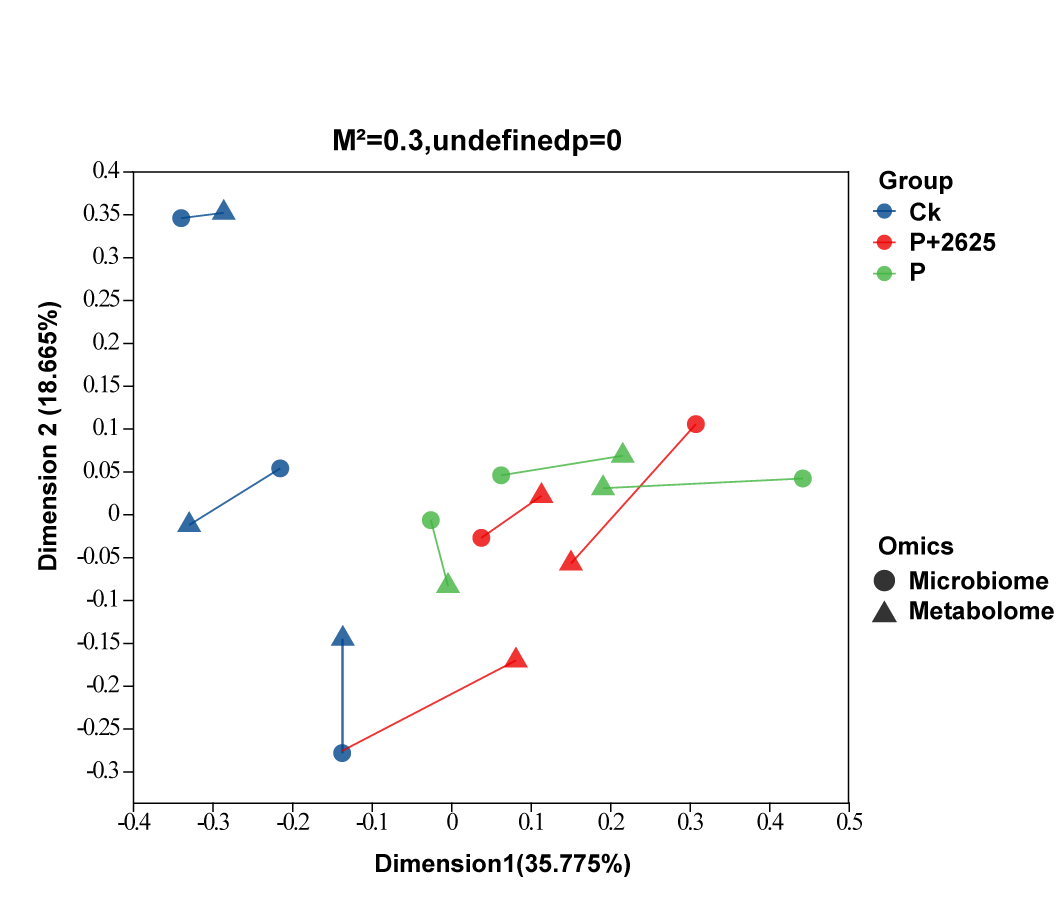

Supplement: SUPPLEMENTARY FIGURE S3 — Procrustes analysis between rhizosphere soil microbiome composition and root exudate metabolomic profiles. [file Image_3.tif]
